# Deregulated Expression of SRC, LYN and CKB Kinases by DNA Methylation and Its Potential Role in Gastric Cancer Invasiveness and Metastasis

**DOI:** 10.1371/journal.pone.0140492

**Published:** 2015-10-13

**Authors:** Adriano Azevedo Mello, Mariana Ferreira Leal, Juan Antonio Rey, Giovanny Rebouças Pinto, Leticia Martins Lamarão, Raquel Carvalho Montenegro, Ana Paula Negreiros Nunes Alves, Paulo Pimentel Assumpção, Barbara do Nascimento Borges, Marília Cardoso Smith, Rommel Rodriguez Burbano

**Affiliations:** 1 Centro de Ciências Biológicas e da Saúde, Universidade Federal de Campina Grande, Campina Grande, PB, Brazil; 2 Disciplina de Genética, Departamento de Morfologia e Genética, Universidade Federal de São Paulo, São Paulo, SP, Brazil; 3 Departamento de Ortopedia e Traumatologia, Universidade Federal de São Paulo, São Paulo, SP, Brazil; 4 Laboratorio de Oncogenética Molecular, Hospital Universitario La Paz, Madrid, Madrid, Spain; 5 Departmento de Biomedicina, Universidade Federal do Piauí, Parnaíba, PI, Brazil; 6 Laboratório de Testes de Ácidos Nucleicos, Fundação Centro de Hemoterapia e Hematologia do Pará, Belém, PA, Brazil; 7 Instituto de Ciências Biológicas, Universidade Federal do Pará, Belém, PA, Brazil; 8 Departamento de Patologia Oral, Faculdade de Odontologia, Universidade Federal do Ceará, Fortaleza, CE, Brasil; 9 Núcleo de Pesquisa em Oncologia, Hospital Universitário João de Barros Barreto, Universidade Federal do Pará, Belém, PA, Brazil; 10 Centro de Tecnologia Agropecuária, Instituto Socioambiental e dos Recursos Hídricos, Universidade Federal Rural da Amazônia, Belém, PA, Brazil; University of Florida, UNITED STATES

## Abstract

Kinases are downstream modulators and effectors of several cellular signaling cascades and play key roles in the development of neoplastic disease. In this study, we aimed to evaluate SRC, LYN and CKB protein and mRNA expression, as well as their promoter methylation, in gastric cancer. We found elevated expression of SRC and LYN kinase mRNA and protein but decreased levels of CKB kinase, alterations that may have a role in the invasiveness and metastasis of gastric tumors. Expression of the three studied kinases was also associated with MYC oncogene expression, a possible biomarker for gastric cancer. To understand the mechanisms that regulate the expression of these genes, we evaluated the DNA promoter methylation of the three kinases. We found that reduced *SRC* and *LYN* methylation and increased *CKB* methylation was associated with gastric cancer. The reduced *SRC* and *LYN* methylation was associated with increased levels of mRNA and protein expression, suggesting that DNA methylation is involved in regulating the expression of these kinases. Conversely, reduced *CKB* methylation was observed in samples with reduced mRNA and protein expression, suggesting CKB expression was found to be only partly regulated by DNA methylation. Additionally, we found that alterations in the DNA methylation pattern of the three studied kinases were also associated with the gastric cancer onset, advanced gastric cancer, deeper tumor invasion and the presence of metastasis. Therefore, SRC, LYN and CKB expression or DNA methylation could be useful markers for predicting tumor progression and targeting in anti-cancer strategies.

## Introduction

Gastric cancer (GC) is the fourth most frequent cancer type and the second highest cause of cancer mortality worldwide [1]. Treatment of GC at advanced stages remains difficult, and the prognosis is still poor, partly as a result of local recurrence, tumor invasion and/or metastasis. The overall relative 5-year survival rate is currently less than 20% [2]. A better understanding of the biology of the progression of this neoplasia is crucial to reducing the mortality rate with the development of novel patient management and therapeutic strategies.

Phosphotransferases, also known as kinases, are downstream modulators and effectors of several cellular signaling cascades and play key roles in the development of neoplastic disease [3]. To date, several protein kinase-interacting drugs have been registered for clinical trials [4]. We previously performed screening to identify kinase proteins expressed in GC using Capture Compound Mass Spectrometry [5, 6] (S1 File), and 22 kinase proteins, including SRC, LYN and CKB, were detected (S1 Table). These three kinases were selected for further investigations (S1 Fig).

SRC was the first proto-oncogene discovered, and it plays a central role in cellular signal transduction pathways. Aberrant SRC activity is observed in several human cancers, including GC [7–9], and it may be important during tumor development and progression [10, 11]. The mitogenic function of SRC is, at least in part, mediated by the induction of MYC, a cell cycle regulator and transcription factor [12, 13]. Our group previously described MYC upregulation in human GC and in N-methyl-nitrosourea-treated non-human primates [14–19]. Because the activation of SRC, as well as that of other kinases, has pleiotropic effects that depend on the cell type and context [20], it is still important to understand the possible relationship between kinases and MYC expression in gastric carcinogenesis and the molecular mechanism involved in their regulation.

LYN is another member of the SRC family of kinases, and the *LYN* gene is located at chromosome 8q13. Our group previously reported the presence of gains of chromosome 8 (on which the *MYC* gene is also located) in GC cases from Northern Brazil [16, 21–23] and in all GC cell lines established from neoplasias in this population [24, 25]. Therefore, this chromosome may contain important genes involved in gastric carcinogenesis. To our knowledge, no previous study has investigated the role of LYN and its regulation in GC. However, LYN overexpression has been reported in several cancers [26–32]. In addition, the regulation of *LYN* by DNA methylation was demonstrated in both colorectal cancer and Ewing’s sarcoma [33, 34], and *LYN* methylation has been observed in some hematopoietic and non-hematopoietic cell lines [35]. DNA methylation is a molecular modification of DNA that is tightly associated with gene function and cell type-specific gene function [36]. Moreover, DNA methylation may be a robust biomarker, as it is vastly more stable than RNA or protein and is therefore a promising target for the development of new approaches for the diagnosis and prognosis of cancers [36].

CKB is one of two cytosolic isoforms of creatine kinase and may participate in metabolic processes involving glycolysis in non-muscle cells [37]. In contrast to normal cells, which primarily generate energy via oxidative phosphorylation, most cancer cells prefer aerobic glycolysis, which is known as the Warburg effect [38]. Interestingly, the MYC oncogene appears to activate several glucose transporters and glycolytic enzymes, thereby contributing to the Warburg effect [39]. Our previous proteomic study revealed that several proteins involved in energy production processes were deregulated in GC samples and reinforced the Warburg effect in this neoplasia [40]. The role of CKB in GC remains poorly understood: some transcriptomic studies reported the upregulation of *CKB* in GC samples [41, 42], whereas another showed *CKB* downregulation [43]. In addition, as for the *LYN* gene, *CKB* methylation was previously described in hematologic and solid cancer cell lines, including GC cell lines [44]. The methylation of *CKB* appears to be related to its reduced level of expression; however, further investigation is still necessary to understand the regulation of *CKB* by epigenetic modifications.

Therefore, we first aimed to evaluate the mRNA and protein expression of SRC, LYN and CKB in a large set of GC samples. Then, we evaluated whether these genes may be regulated by DNA methylation in gastric carcinogenesis. In addition, we investigated the possible association between kinase expression or methylation and clinical variables as well as MYC expression and methylation.

## Material and Methods

### Tissue samples

Kinase expression and methylation patterns were evaluated in 138 pairs of GC samples and their corresponding non-neoplastic gastric tissue samples obtained from patients who underwent gastrectomy in Northern Brazil. All of the patients had negative histories of exposure to either chemotherapy or radiotherapy prior to surgery, and there was no co-occurrence of other diagnosed cancers. This study was approved by the ethics committee of João de Barros Barreto University Hospital (Protocol #316737). Written informed consent with approval of the ethics committee was obtained from all patients prior to specimen collection.

Part of each dissected tumor sample was formalin-fixed and paraffin-embedded (FFPE). Sections of FFPE tissue were stained with hematoxylin-eosin for histological evaluation or used for immunohistochemistry (IHC) analysis. Additional portions of each tumor and paired non-neoplastic tissue specimens were snap-frozen in liquid nitrogen and stored at -80°C until protein and nucleic acid purification.

All of the samples were classified according to Laurén [45], and the tumors were staged according to the TNM staging criteria [46]. The presence of *Helicobacter pylori*, a class I carcinogen, in gastric samples was detected by the rapid urease test, and its virulence factor cytotoxicity associated gene A (CagA gene) was detect by PCR using DNA purified simultaneously with proteins and mRNA, as previously performed by our group [47]. Epstein-Barr virus (EBV) was detected by RNA in situ hybridization [47].

For 49 of these pairs of neoplastic and non-neoplastic samples, we assessed the MYC immunoreactivity, mRNA expression and methylation status data previously published by our group [18].

### Protein, mRNA and DNA purification

Total protein, mRNA, and DNA were simultaneously isolated from gastric tissue samples using the AllPrep DNA/RNA/Protein Kit (Qiagen, Germany) according to the manufacturer's instructions. The protein pellet was dissolved in a buffer containing 7 M urea, 2 M thiourea, 4% CHAPS, 50 mM DTT, 1% Protease Inhibitor Cocktail (Sigma-Aldrich, USA), and 0.5% each Phosphatase Inhibitor Cocktail 1 and 2 (Sigma-Aldrich, USA), as previously performed by our group [48]. The protein concentrations were determined by the method of Bradford (Sigma-Aldrich, USA). The RNA concentration and quality were determined using a NanoDrop spectrophotometer (Kisker, Germany) and 1% agarose gels, respectively. Samples were stored at -80°C until use.

### Protein immunoreactivity analysis

Tumor tissue sections (3 or 4-mm thick) were deparaffinized in xylene and rehydrated in a graded series of ethanol. After heat-induced epitope retrieval, the tissue sections were incubated with primary mouse monoclonal antibodies against SRC (dilution 1:400; clone 28, Life Technologies, USA), LYN (dilution 1:400; clone C13F9; Life Technologies, USA) or CKB (dilution 1:250; HPA001254, Santa Cruz Biotechnology, USA). A universal peroxidase-conjugated secondary antibody kit (LSAB System, DakoCytomation, USA) was used for detection. We used 3,30-diamino-benzidine/H_2_O_2_ (DakoCytomation, Denmark) as the chromogen and hematoxylin as the counterstain. A protein immunoreactivity-positive sample was defined as one having 10% or more neoplastic cells that were positive for the protein.

### Protein expression analysis

Western blot analysis was performed as previously described by our group [49]. Reduced protein (25 μg) from each sample was separated by 12.5% homogeneous SDS-PAGE and electro-blotted onto a PVDF membrane (Hybond-P, GE Healthcare, USA). The PVDF membrane was blocked with phosphate-buffered saline containing 0.1% Tween 20, and 5% low fat milk and incubated overnight at 4°C with the corresponding primary antibodies: anti-SRC (dilution 1:1000; clone 28, Life Technologies, USA), anti-LYN (dilution 1:1000; clone C13F9; Life Technologies, USA), anti-CKB (dilution 1:400; HPA001254, Santa Cruz Biotechnology, USA), and anti-ACTB (dilution 1:250; Ac-15, Life Technologies, USA). After extensive washing, a peroxidase-conjugated secondary antibody was added for 1 h at room temperature. Immunoreactive bands were visualized using the western blotting Luminol reagent, and the images were acquired using an ImageQuant 350 digital image system (GE Healthcare, Sweden). ACTB was used as a loading reference control.

### mRNA expression analysis

First, RNA was reverse-transcribed using the High-Capacity cDNA Archive kit according to the manufacturer’s protocol (Life Technologies, USA). Complementary DNA was then amplified by real-time reverse transcription quantitative PCR (RT-qPCR) using TaqMan probes purchased as Assays-on-demand Products for Gene Expression (Life Technologies, USA) and a 7500 Fast Real-Time PCR instrument (Life Technologies, USA). The *GAPDH* gene was selected as an internal control for RNA input and reverse-transcription efficiency. All RT-qPCRs were performed in triplicate for both the target genes (*SRC*: Hs01082246_m1; *LYN*: Hs00176719_m1; *CKB*: Hs00176484_m1) and the internal control (*GAPDH*: NM_002046.3).

The relative quantification of gene expression was calculated according to Livak and Schmittgen [50]. The corresponding control sample was designated as a calibrator from each tumor.

### DNA methylation analysis

The methylation pattern and frequency of kinase genes were evaluated by methylation-specific PCR (MSP) [51]. The EZ DNA Methylation-Lightning™ Kit (Zymo Research, USA) was used to modify the gDNA by bisulfite treatment, converting unmethylated cytosines into uracils and leaving methylated cytosines unchanged. Specific primers for the gene promoters are described in Table 1.

**Table 1:** Primer sequences (5’-3’) for methylation analysis.

Gene	Type	Sense	Antisense	Anneling temperature (°C)	Product size (bp)	CpG sites analyzed
*SRC*	MSP—M	5' GATTATTTTGGCGTCGGATC 3'	5' ATCACAACAAAAAACCGCG 3'	58	141	4
	MSP—U	5' GGATTATTTTGGTGTTGGATT 3'	5' CATCACAACAAAAAACCACA 3'	54	141	4
	BSP	5' GTGGGGTGTTTAGTTTTAAAAGG 3'	5' TCCTAACCACCACCTAACCTAA 3'	56	447	8
*LYN*	MSP—M	5' AGGTTTCGTAGGTGTTCGTC 3'	5' CGACTTCCCCACTATATACGA 3'	55	152	4
	MSP—U	5' TTGAGGTTTTGTAGGTGTTTGTT 3'	5' CAACTTCCCCACTATATACAAAAA 3'	55	152	4
	BSP	5' GTTTTTTGGTAGTGGGAGATG 3'	5' AAAAATACCACCATAAACCCAA 3'	55	298	21
*CKB*	MSP—M	5' CGTTAAGGGATTGGGTTTC 3'	5' ATAAAATCCCAACGACGAAA 3'	56	164	4
	MSP—U	5' GTGTGTTAAGGGATTGGGTTTT 3'	5' ATAAAATCCCAACAACAAAAAAA 3'	56	164	4
	BSP	5' TGGAGTTTTTTGTTTTTTTTTTT 3'	5' CTCAAAACATACCCAAAAAAAA 3'	54	345	15

MSP: primers for methyl-specific PCR; BSP: primers for Bisulfite Sequencing PCR; M: primer for methylated sequence by MSP; U: primer for unmethylated sequences by MSP; bp: base pair.

PCR reactions were carried out using 0.1 μmol/L dNTPs, 2 μmol/L MgCl_2_, 0.5 μmol primers, 1.25 U Taq DNA polymerase, and 100 ng bisulfite-modified DNA. After initial denaturation for 5 min at 94°C, 40 cycles at 94°C for 45 s, the annealing temperature (Table 1) for 45 s, and 72°C for 30 s were carried out, followed by a final extension for 5 min at 72°C. The PCR products were directly loaded onto 3% agarose gels and electrophoresed. The gel was stained with SYBR^®^ Safe DNA Gel Stain (Life Technologies, USA) and directly visualized under UV illumination. As a positive control for all MSP reactions, a gDNA sample was completely methylated using CpG Methylase (SssI, New England Biolabs, USA) following the manufacturer’s instructions. Furthermore, primers for detecting the wild-type sequence were used to monitor the complete conversion of DNA obtained in the bisulfite reaction.

The samples were stratified as follows: 1) a sample was defined as hypomethylated when a positive amplification product was detected only in the PCR with specific primers for unmethylated sequences; 2) a sample was defined as hypermethylated when positive amplification was detected only in the PCR with specific primers for methylated sequences; 3) a sample was defined as partially methylated when positive amplification was detected in the PCR with the two primer sets.

The primers’ specificity and MSP results were confirmed using a bisulfite sequencing PCR (BSP) approach [52]. BSP was also used to evaluate the percentage of methylation. The primers and anneling temperatures of BSP are described in Table 1. Following amplification, the fragments were purified using the NucleoSpin Gel and PCR Clean-up Kit (Macherey-Nagel, Germany), ligated into pGEM T-easy Vector (Promega, Germany) and cloned into competent *E*. *coli* JM109 cells. After incubation time, white colonies were selected for PCR with M13 forward (5'-TGTAAAACGACGGCCAGT-3') and M13 reverse (5'-CAGGAAACAGCTATGAC-3') primers [53]. After initial denaturation for 3 min at 94°C, the PCR amplification consisted of 35 cycles at 94°C for 30 s, 55°C for 30 s, and 72°C for 90 s were carried out, followed by a final extension for 5 min at 72°C. The PCR products were visualized in agarose gel. Six clones were selected for purification and sequenced in an ABI310 automatic sequencer (Applied Biosystems, Foster City, CA, USA). All sequences were aligned with BioEdit v7.0.5 [54], and the methylation analyses were performed with the BiQ Analyzer software [55]. The percentage of methylation for each sample was calculated by dividing the number of methylated CpGs by the total number of CpGs sequenced (CpGs in all the six clones).

### Statistical analyses

The data are shown as the frequency, median and interquartile range (IQR). The Shapiro-Wilk test was used to evaluate the distribution of the age, mRNA, protein expression and percentage of methylation data and to determine the appropriate subsequent test for statistical comparisons. The Mann-Whitney test was used to investigate possible associations between kinase mRNA or protein expression and categorical variables, such as immunoreactivity, methylation pattern and clinicopathological features. The Mann-Whitney test was used to investigate possible associations between the percentage of methylation and immunoreactivity and clinicopathological features. Wilcoxon test was used to compare the percentage of methylation between pairs of neoplastic and non-neoplastic samples. An association between categorical variables was analyzed using the Chi-squared (χ^2^) test. A Spearman correlation test was used to evaluate the possible correlation between mRNA and protein expression, as well as promoter methylation. A p-value less than 0.05 was considered significant. Bonferroni adjustment of the p-value was applied when multiple comparisons were performed, with the alpha level being divided by the number of comparisons.

## Results

### Kinase expression in gastric tumors

**Fig 1:**
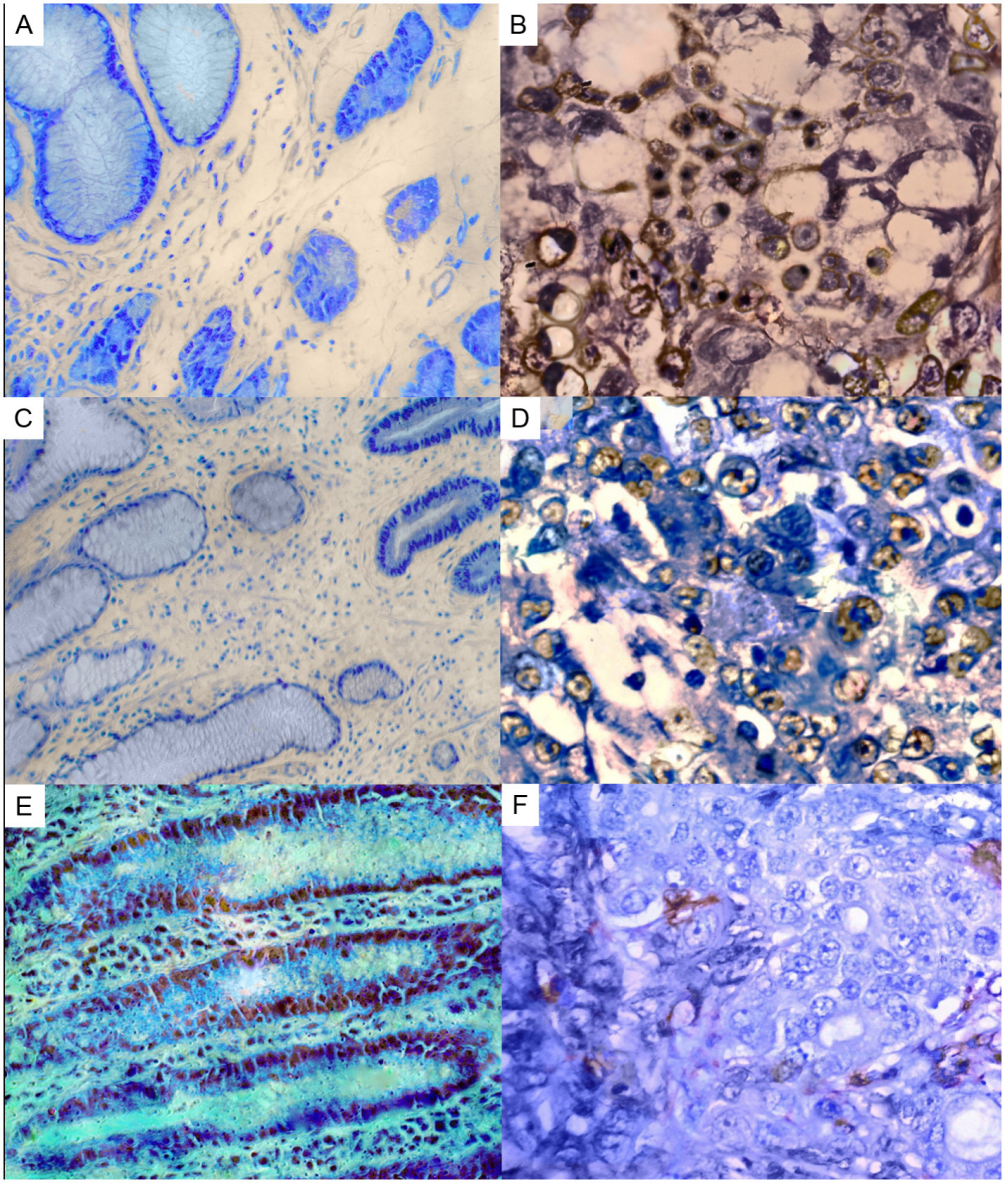
Kinases immunoreactivity in gastric tissue samples. A) gastric mucosa without SRC immunoreactivity; B) diffuse-type gastric cancer presenting cell membrane and cytoplasmic immunoreactivity of SRC; C) non-neoplastic gastric tissue without LYN immunoreactivity; D) intestinal-type gastric cancer presenting LYN immunoreactivity; E) non-neoplastic gastric mucosa showing weak cytoplasmic CKB staining in glandular cells; F) diffuse-type gastric cancer cells without CKB immunoreactivity.

Non-atypical gastric cells did not present SRC or LYN immunoreactivity (Fig 1A and 1C). However, SRC immunoreactivity was observed in dysplastic cells. Cell membrane and cytoplasmic immunoreactivity for SRC and LYN was detected in neoplastic cells (Fig 1B and 1D), and LYN also presented nucleic immunoreactivity. CKB immunoreactivity was detected in the cytoplasm or in the cell membrane in non-neoplastic gastric cells (Fig 1E). In contrast, GC cells did not present CKB immunoreactivity (Fig 1F).

**Fig 2:**
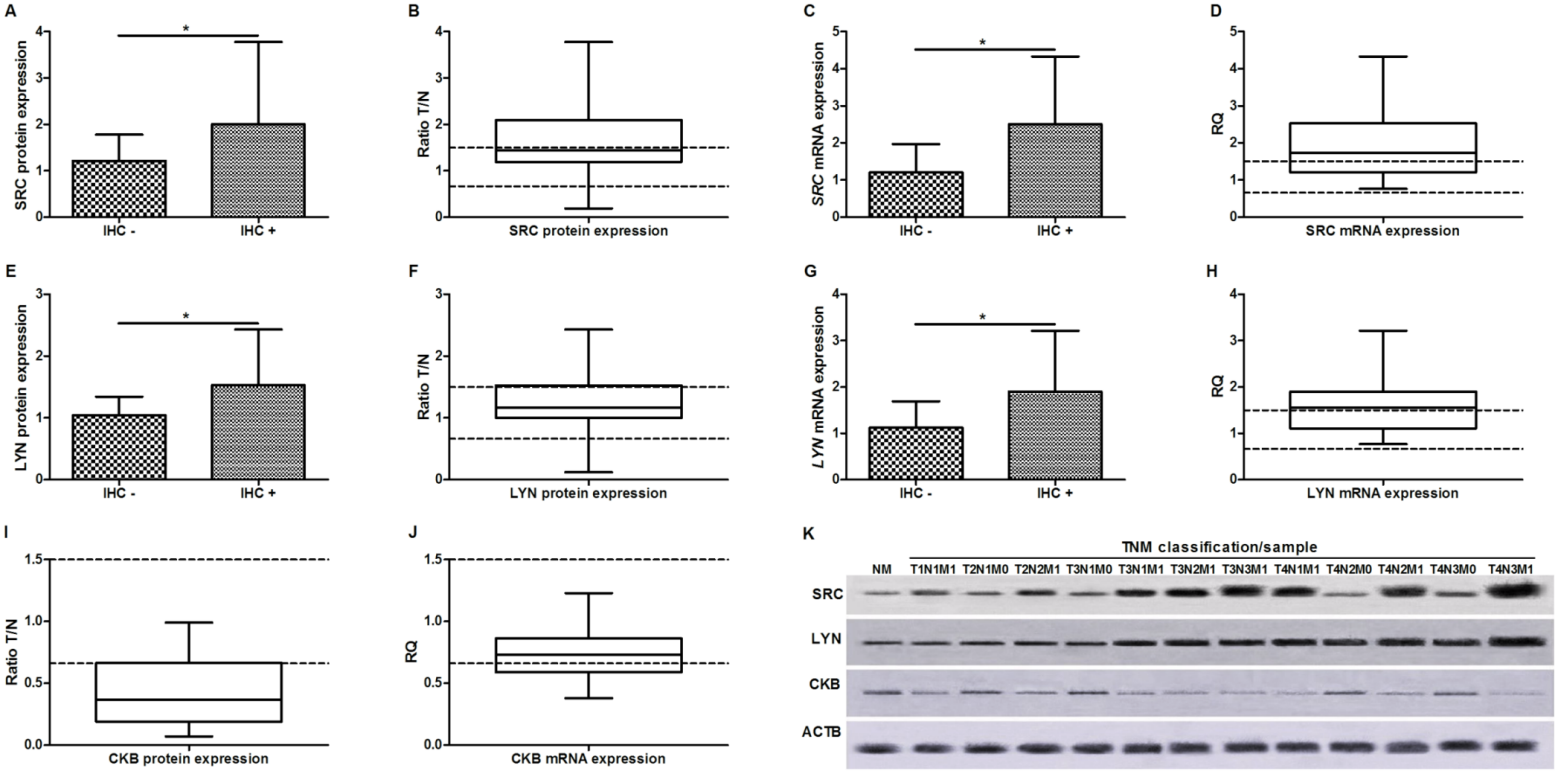
Kinases expression in gastric cancer. A) Association between SRC immunoreactivity and its protein expression; B) SRC protein expression; C) Association between SRC immunoreactivity and its mRNA expression; D) *SRC* mRNA expression. E) Association between LYN immunoreactivity and its protein expression; F) LYN protein expression; G) Association between LYN immunoreactivity and its mRNA expression; H) *LYN* mRNA expression; I) CKB protein expression; J) *CKB* mRNA expression; K) representative image of Western-blot, in each TNM of each sample is show. Protein and mRNA expression were determined by Western-blot and RT-qPCR analysis, respectively. In all graphs, the expression in gastric tumors was normalized by matched non-neoplastic gastric tissue. *Significant difference between groups by Mann-Whitney (p < 0.05). IHC+: cases presenting protein immunoreactivity; IHC–: cases without protein immunoreactivity; NM: normal mucosa sample.

SRC, LYN and CKB immunoreactivity was detected in 72 (52.2%), 66 (47.8%) and 0 (0%) of the tumor samples. SRC and LYN immunoreactivity were associated with higher mRNA and protein levels in GC samples (p < 0.001, for all comparisons; Mann-Whitney test; Fig 2A, 2C, 2E and 2G). The protein and mRNA levels of SRC were increased at least 1.5-fold (at least a 50% increase in expression) in 67 (48.6%) and 80 (58%), respectively, GC samples in relation to their matched non-neoplastic gastric samples (Fig 2B, 2D and 2K). Moreover, the protein and mRNA levels of LYN were increased at least 1.5-fold in 36 (26.1%) and 72 (52.2%) GC samples, respectively (Fig 2F, 2H and 2K). Conversely, downregulation of CKB protein and mRNA (at least 50% decrease of expression) was detected in 104 (75.4%) and 49 (35.5%) GC samples, respectively (Fig 2I, 2J and 2K). A strong and direct correlation was observed between mRNA and protein expression for SRC (p < 0.001, ρ = 0.856, Spearman correlation test), LYN (p < 0.001, ρ = 0.762) and CKB (p < 0.001, ρ = 0.819).

The immunoreactivity of SRC was associated with the immunoreactivity of LYN (p < 0.001, χ^2^ test), with 52 (37.7%) of the GC samples presenting immunoreactivity for both proteins. In addition, a direct correlation was observed between SRC and LYN protein (p < 0.001, ρ = 0.556) and mRNA (p < 0.001, ρ = 0.779) expression. The levels of CKB protein and mRNA expression were inversely correlated with SRC (p < 0.001, ρ = -0.734; p < 0.001, ρ = -0.806, respectively) and LYN (p < 0.001, ρ = -0.643; p < 0.001, ρ = -0.703, respectively).

Table 2 shows the results for SRC, LYN and CKB expression and the clinicopathological characteristics. The tumors of patients with late-onset GC presented significantly higher SRC and LYN protein (by IHC and western blotting) and mRNA (by RT-qPCR) expression, as well as reduced CKB protein expression by western blotting, compared with early-onset CG samples (p < 0.05, for all comparisons; Table 2). Increased protein and mRNA expression of SRC and LYN and reduced CKB expression were associated with advanced stage, deeper tumor invasion, and the presence of lymph node and distant metastases (p < 0.05, for all comparisons; Table 2).

**Table 2:** Clinicopathological variables and kinases expression in gastric cancer.

Variable	N	SRC immunoreactivity		SRC protein		*SRC* mRNA		LYN immunoreactivity		LYN protein		*LYN* mRNA		CKB immunoreactivity		CKB protein		*CKB* mRNA	
		N (%) of positive cases	p-value^a^	Ratio T/N [median (IQR)]	p-value^b^	RQ [median (IQR)]	p-value^b^	N (%) of positive cases	p-value^a^	Ratio T/N [median (IQR)]	p-value^b^	RQ [median (IQR)]	p-value^b^	N (%) of negative cases	p-value^a^	Ratio T/N [median (IQR)]	p-value^b^	RQ [median (IQR)]	p-value^b^
**Gender**																			
Female	50	27 (54)	0.442	1.33 (0.82)	0.285	1.63 (1.16)	0.674	25 (50)	0.417	1.19 (0.48)	0.728	1.56 (0.77)	0.910	50 (100)	-	0.37 (0.51)	0.529	0.73 (0.26)	0.750
Male	88	45 (51.1)		1.67 (0.99)		1.79 (1.36)		41 (62.1)		1.15 (0.53)		1.56 (0.82)		88 (100)		0.36 (0.47)		0.73 (0.31)	
**Onset**																			
< 45 years	35	13 (37.1)	0.031*	1.29 (0.69)	0.011*	1.46 (1.05)	0.027*	10 (28.6)	0.007*	1.08 (0.27)	0.008*	1.23 (0.64)	0.014*	35 (100)	-	0.49 (0.48)	0.028*	0.77 (0.22)	0.132
≥ 45 years	103	59 (57.3)		1.67 (1.02)		1.82 (1.5)		56 (54.4)		1.22 (0.62)		1.64 (0.82)		103 (100)		0.32 (0.49)		0.71 (0.30)	
**Tumor location**																			
Cardia	52	22 (42.3)	0.052	1.35 (0.77)	0.263	1.46 (1.2)	0.152	21 (40.4)	0.118	1.14 (0.57)	0.276	1.25 (0.82)	0.090	52 (100)		0.40 (0.47)	0.190	0.74 (0.24)	0.309
Non-cardia	82	50 (58.1)		1.64 (0.94)		1.85 (1.35)		45 (52.3)		1.19 (0.50)		1.62 (0.79)		86 (100)		0.34 (0.47)		0.72 (0.28)	
**Histological type**																			
Diffuse	64	35 (54.7)	0.353	1.58 (0.84)	0.665	1.89 (1.34)	0.838	31 (48.4)	0.515	1.18 (0.55)	0.785	1.58 (0.82)	0.629	64 (100)	-	0.33 (0.48)	0.651	0.71 (0.26)	0.210
Intestinal	74	37 (50)		1.44 (1.03)		1.63 (1.40)		35 (47.3)		1.15 (0.45)		1.50 (0.80)		74 (100)		0.39 (0.50)		0.74 (0.27)	
**Stage**																			
Early	12	3 (25)	0.046*	1.04 (0.67)	0.001*	1.04 (0.78)	0.002*	0 (0)	<0.001*	1.02 (0.19)	0.003*	0.89 (0.27)	<0.001*	12 (100)		0.74 (0.58)	0.015*	0.95 (0.38)	0.019*
Advanced	126	69 (54.8)		1.55 (0.94)		1.80 (1.34)		66 (52.4)		1.21 (0.53)		1.61 (0.77)		126 (100)		0.36 (0.47)		0.72 (0.26)	
**Tumor invasion**																			
T1/T2	43	14 (32.6)	0.002*	1.24 (0.64)	<0.001*	1.29 (0.99)	<0.001*	5 (11.6)	<0.001*	1.05 (0.26)	<0.001*	1.17 (0.30)	<0.001*	43 (100)	-	0.66 (0.54)	<0.001*	0.88 (0.31)	<0.001*
T3/T4	95	58 (61.1)		1.76 (1.07)		1.99 (1.55)		61 (64.2)		1.32 (0.60)		1.75 (0.81)		95 (100)		0.28 (0.40)		0.69 (0.24)	
**Lymph node metastasis**																			
Absent	16	0 (0)	<0.001*	1.13 (0.26)	<0.001*	0.93 (0.24)	<0.001*	1 (6.3)	<0.001*	1.01 (0.26)	<0.001*	0.99 (0.17)	<0.001*	16 (100)	-	0.73 (0.20)	<0.001*	0.95 (0.24)	<0.001*
Present	122	72 (59)		1.67 (0.98)		1.89 (1.29)		65 (53.3)		1.23 (0.52)		1.63 (0.73)		122 (100)		0.31 (0.40)		0.70 (0.25)	
**Distant metastasis**																			
Absent	70	11 (15.7)	<0.001*	1.22 (0.25)	<0.001*	1.22 (0.41)	<0.001*	16 (22.9)	<0.001*	106 (0.28)	<0.001*	1.11 (0.38)	<0.001*	70 (100)	-	0.66 (0.31)	<0.001*	0.86 (0.22)	<0.001*
Present	68	61 (89.7)		2.11 (0.76)		2.53 (0.92)		50 (73.5)		1.43 (0.61)		1.81 (0.44)		68 (100)		0.19 (0.12)		0.60 (0.16)	
***H*. *pylori***																			
Negative	14	9 (64.3)	0.251	1.47 (1.03)	0.647	1.71 (1.47)	0.764	9 (64.3)	0.154	1.20 (0.53)	0.838	1.60 (0.91)	0.762	14 (100)	-	0.30 (0.34)	0.391	0.73 (0.18)	0.841
Positive	124	63 (50.8)		1.44 (0.90)		1.73 (1.32)		57 (46)		1.16 (0.52)		1.54 (0.79)		124 (100)		0.37 (0.50)		0.73 (0.29)	
**CagA**																			
Negative	49	26 (53.1)	0.510	1.55 (0.84)	0.730	1.76 (1.17)	0.779	24 (49)	0.490	1.15 (0.52)	0.304	1.41 (0.73)	0.441	49 (100)	-	0.34 (0.44)	0.836	0.74 (0.30)	0.437
Positive	89	46 (51.7)		1.44 (0.99)		1.70 (1.40)		42 (47.2)		1.17 (0.49)		1.58 (0.82)		89 (100)		0.37 (0.51)		0.73 (0.28)	
**EBV**																			
Negative	117	60 (51.3)	0.399	1.44 (0.81)	0.259	1.67 (1.22)	0.371	55 (47)	0.414	1.15 (0.53)	0.718	1.56 (0.79)	0.652	117 (100)	-	0.37 (0.49)	0.397	0.73 (0.29)	0.543
Positive	21	12 (57.1)		1.77 (1.21)		2.24 (1.67)		11 (52.4)		1.18 (0.56)		1.53 (0.95)		21 (100)		0.27 (0.51)		0.70 (0.28)	

^a^p-value by χ^2^ test

^b^p-value by Mann-Whitney test

*Significantly difference between groups (p < 0.05)

N: number of samples; IQR: interquartile range; EBV: *Epstein-Barr virus*

**Fig 3:**
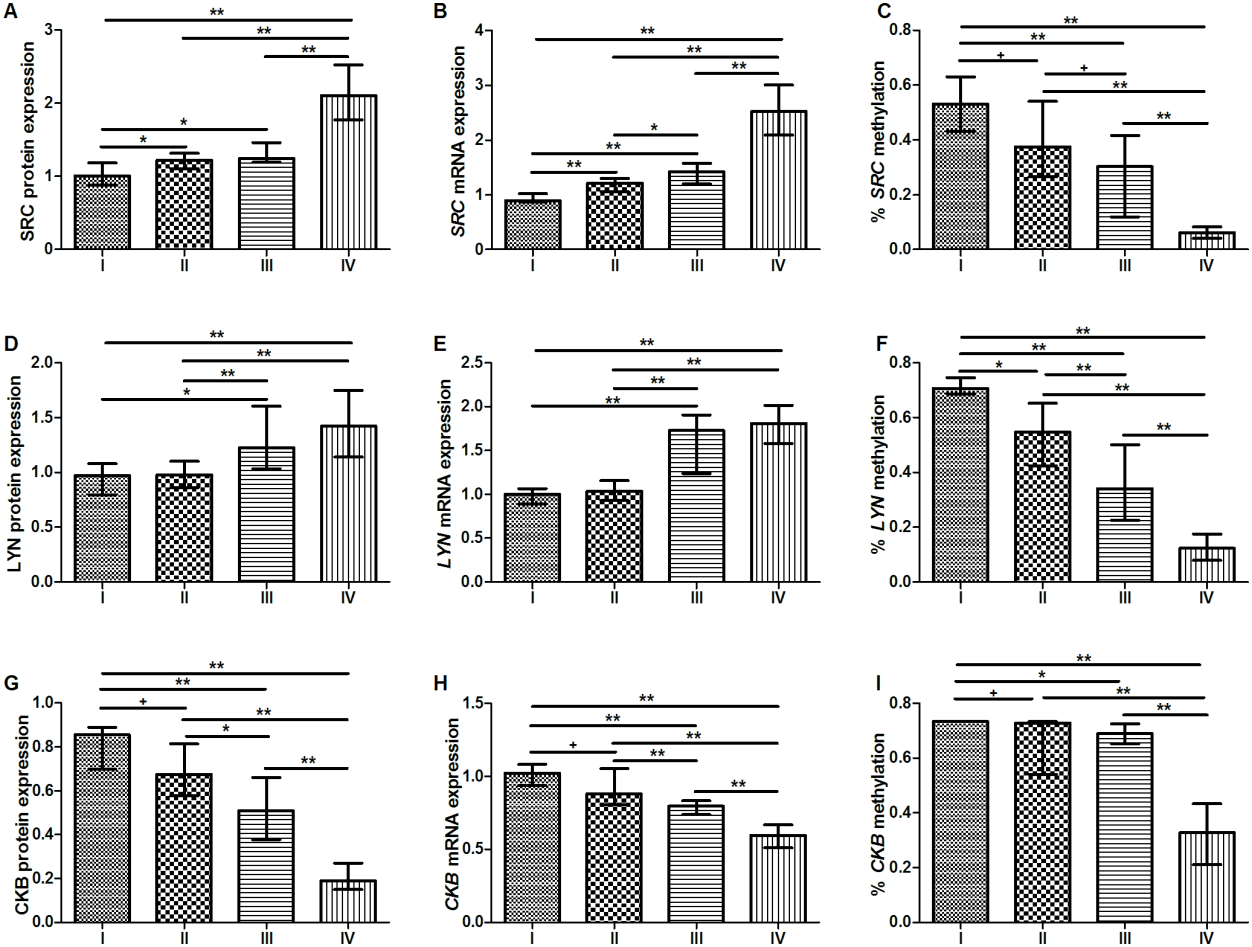
Kinases protein and mRNA expression and DNA methylation by tumor stage. A) SRC protein expression; B) *SRC* mRNA expression; C) Percentage of *SRC* methylation; D) LYN protein expression; E) *LYN* mRNA expression; F) Percentage of *LYN* methylation; G) CKB protein expression; H) *CKB* mRNA expression; I) Percentage of *CKB* methylation. Protein and mRNA expression were determined by Western-blot and RT-qPCR analysis, respectively. In these expression analyses, the expression in gastric tumors was normalized by matched non-neoplastic gastric tissue. DNA methylation was determined by bisulfite sequencing PCR. *Significant difference between groups by Mann-Whitney test followed by Bonferroni corrections for multiple comparison analysis (p < 0.008); **Significant difference between groups by Mann-Whitney test followed by Bonferroni corrections for multiple comparison analysis (p < 0.001); ^+^Difference between groups but not statistically significant after Bonferroni adjustment (p < 0.05).

A gradual significant increase in SRC protein (by western blotting) and mRNA expression was observed corresponding to the tumor stage (p < 0.008, for most of the comparisons; Mann-Whitney test followed by Bonferroni correction; Fig 3A and 3B). In contrast, a gradual significant decrease in CKB protein and mRNA expression was observed corresponding to the tumor stage (p < 0.008, for most of the comparisons; Fig 3G and 3H). With regard to LYN expression, we did not observe a significant difference between stages I and II or between stages III and IV. However, stages I and II were significantly different from stages III and IV (p < 0.008, for these comparisons; Fig 3D and 3E).

### Kinase gene methylation patterns in gastric samples

**Fig 4:**
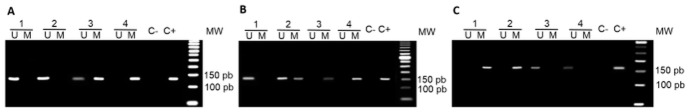
Methylation analysis of the kinases promoters showing methylated and unmethylated bands. A) *SRC* promoter methylation analysis, in which samples 1 and 2 presented hypomethylated promoter, sample 3 presented partial methylation and sample 4 presented hypermethylated promoter; B) *LYN* promoter methylation analysis, in which samples 1 presented hypomethylated promoter, sample 2 presented partial methylation and samples 3 and 4 presented hypermethylated promoter; C) *CKB* promoter methylation analysis, in which samples 1 and 2 presented hypermethylated promoter, and samples 3 and 4 presented hypomethylated promoter. C–: blank; C+: positive control, gDNA sample completely methylated; U: PCR with unmethylated primer set; M: PCR with methylated primer set; MW: molecular weight marker; bp: base pairs.

Table 3 shows the methylation pattern of the studied protein kinases in neoplastic and non-neoplastic gastric samples by MSP. Approximately 60% and 30% of the GC samples presented positive amplification with only the unmethylated primer set (hypomethylated samples) for the *SRC* and *LYN* genes, respectively (Fig 4A and 4B). Hypomethylation of these genes was not observed in any non-neoplastic sample. Therefore, the frequency of *SRC* and *LYN* hypomethylation was significantly higher in GC than in non-neoplastic gastric samples (p < 0.001, for all comparisons; χ^2^ test followed by Bonferroni corrections).

**Table 3:** Protein kinases methylation pattern in gastric samples.

Methylation pattern	*SRC*			*LYN*			*CKB*		
	Neoplastic	Non-neoplastic	p-value	Neoplastic	Non-neoplastic	p-value	Neoplastic	Non-neoplastic	p-value
Hypermethylated	26 (18.8)	59 (42.8)	<0.001 ^a^ *	46 (33.6)	82 (59.4)	<0.001 ^a^ *	48 (39)	0 (0)	<0.001 ^a^ *
Partial-methylated	31 (22.5)	79 (57.2)	0.8355 ^b^	50 (36.5)	56 (40.6)	0.1084 ^b^	68 (55.3)	59 (42.8)	<0.001 ^b^ **
Hypomethylated	81 (58.7)	0 (0)	<0.001^c^,^d^ **	41 (29.9)	0 (0)	<0.001^c^,^d^ **	7 (5.7)	79 (91.9)	<0.001 ^c^,^d^ **

^a^p-value of χ^2^ test

^b^p-value of the post-hoc comparison between tissue samples hypermethylated and partial-methylated

^c^p-value of the post-hoc comparison between tissue samples partial-methylated and hypomethylated

^d^p-value of the post-hoc comparison between tissue samples hypermethylated and hypomethylated

*Statistically significant difference between neoplastic and non-neoplastic samples by χ^2^ test (p < 0.05)

**Statistically significant difference between groups by χ^2^ test followed Bonferroni adjustment (p < 0.016)

**Fig 5:**
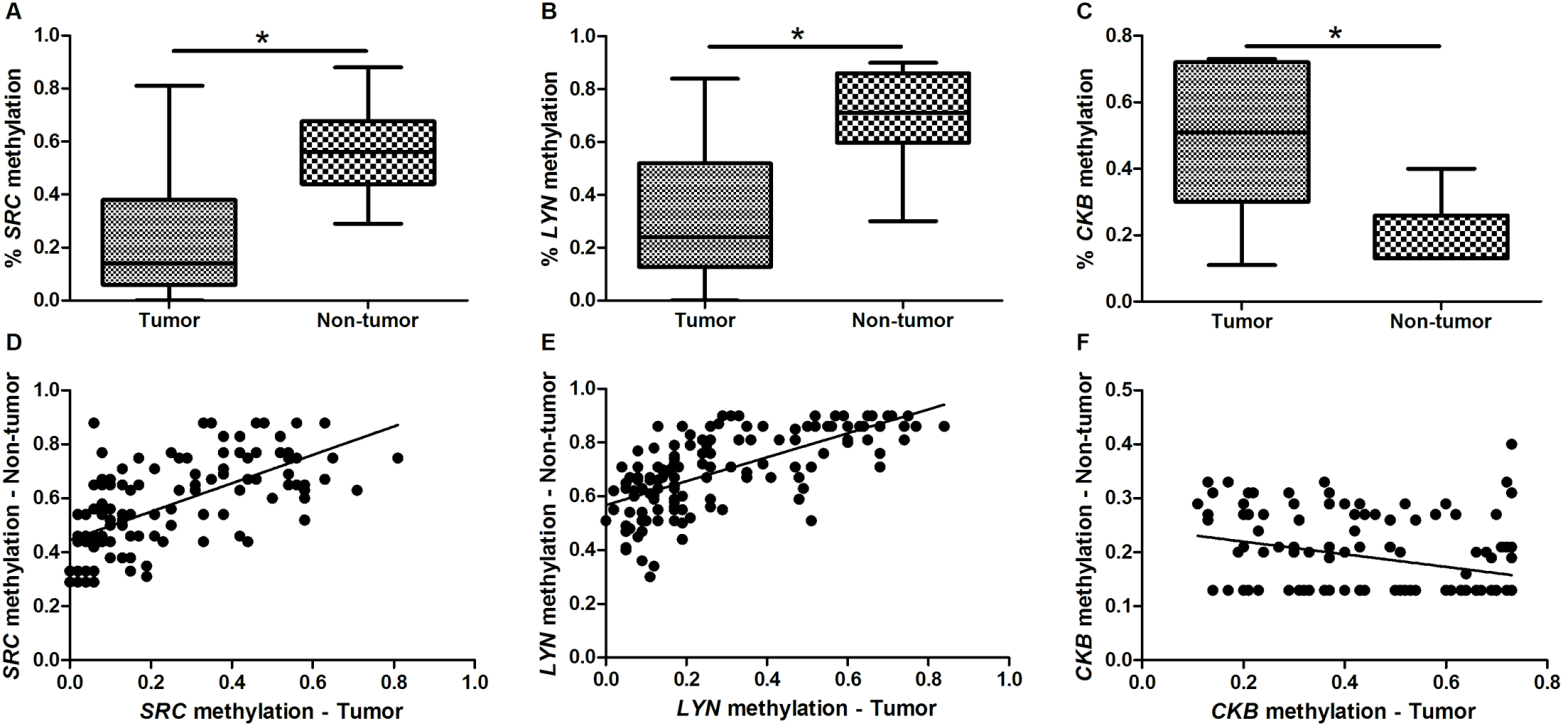
Percentage of methylation in gastric samples. A) *SRC* methylation in gastric tumors and non-tumor samples; B) *LYN* methylation in gastric tumors and non-tumor samples; C) *CKB* methylation in gastric tumors and non-tumor samples; D) Correlation between the percentage of *SRC* methylation in gastric tumors and paired non-tumor samples; E) Correlation between the percentage of *LYN* methylation in gastric tumors and paired non-tumor samples; F) Correlation between the percentage of *CKB* methylation in gastric tumors and paired non-tumor samples. *Significant difference between groups by Mann-Whitney test (p < 0.05).

The BSP analysis confirmed the MSP analysis. By BSP, 82 (59.4%) of neoplastic samples and 0 (0%) of non-neoplastic samples presented a cloned sequence without CpG methylation in *SRC* promoter. In addition, 4 (2.89%) and 1 (0.72%) of neoplastic and non-neoplastic samples presented a cloned sequence without CpG methylation in *LYN* promoter, respectively. By BSP, the percentage of *SRC* [0.135 (0.31) *versus* 0.563 (0.23); p < 0.001] and *LYN* [0.238 (0.40) *versus* 0.7063 (0.26); p < 0.001] methylation was lower in neoplastic samples than in non-neoplastic samples (Fig 5A and 5B).

The *SRC* and *LYN* methylation patterns of the neoplastic and non-neoplastic samples whereas found to be associated (p < 0.001, for both analyses; χ^2^ test). We observed that 70/81 (86.4%) of tumors with hypomethylated *SRC* presented partial methylation of this gene in the matched non-neoplastic sample. In addition, we found that 37/41 (90.24%) of tumors with hypomethylated *LYN* presented partial methylation of this gene in the matched non-neoplastic sample. *SRC* and *LYN* partial methylation in non-neoplastic samples was more frequently observed in individuals presenting tumor samples with hypomethylation of this gene compared with tumors with partial methylation (p < 0.001, for both analyses) or hypermethylation (p < 0.001, for both analyses). Furthermore, partially methylated *LYN* in non-neoplastic samples was also more frequently detected in individuals presenting tumor samples with partial methylation of this gene compared with tumors with hypermethylation (p = 0.004). By BSP, we also observed that the percentage of *SRC* (p < 0.001, ρ = 0.6902; Fig 5D) and *LYN* (p < 0.001, ρ = 0.739; Fig 5E) methylation of neoplastic and non-neoplastic samples were correlated.

*CKB* partial and hypomethylation was observed in both neoplastic and non-neoplastic samples. However, 48 (39%) of GC samples presented *CKB* hypermethylation (Fig 4C), which was not detect in the non-neoplastic samples. Moreover, the frequency of *CKB*-hypermethylated samples was significantly higher in neoplastic compared to non-neoplastic gastric samples (p < 0.001), and *CKB* partial methylation was also significantly more frequent in GC than in non-neoplastic samples (p < 0.001). By BSP, the percentage of *CKB* methylation was higher in neoplastic samples than in non-neoplastic samples [0.511 (0.42) *versus* 0.133 (0.12); p < 0.001; Fig 5C]. Cloned sequences without CpG methylation was detected in 4 (2.89%) and 0 (0%) of neoplastic and non-neoplastic samples, respectively.

The *CKB* methylation pattern of the neoplastic and non-neoplastic samples appeared to be associated (p = 0.014, by χ^2^ test). A 2x2 analysis using the χ^2^ test revealed that pairs in which the tumor samples presented hypermethylated *CKB* and the matched non-neoplastic samples presented hypomethylation of this gene were more frequent than pairs of tumors with hypermethylation and matched non-neoplastic samples with partial methylation (p = 0.0381), but this finding did not reach statistical significance if the Bonferroni adjustment was applied (adjusted α = 0.05/3 = 0.0167). However, by BSP, we observed that the percentage of *CKB* methylation of neoplastic and non-neoplastic samples were inversely correlated (p < 0.001, ρ = -0.375; Fig 5F).

A direct correlation was observed between the *SRC* and *LYN* methylation patterns in the non-neoplastic samples (p < 0.001, ρ = 0.627). In addition, an inverse correlation was detected between *SRC* and *CKB* (p< 0.001, ρ = -0.467) and *LYN* and *CKB* (p < 0.001, ρ = -0.359) methylation. However, in GC samples, a direct correlation was observed among the methylation percentage of the three studied kinases: *SRC* and *LYN* (p < 0.001, ρ = 0.840); *SRC* and *CKB* (p < 0.001, ρ = 0.684); *LYN* and *CKB* (p < 0.001, ρ = 0.663).

### Methylation regulation of kinases

To elucidate the epigenetic regulation of the studied genes, we evaluated the possible association between the promoter methylation and protein immunoreactivity and mRNA and protein expression (by western blotting).

**Fig 6:**
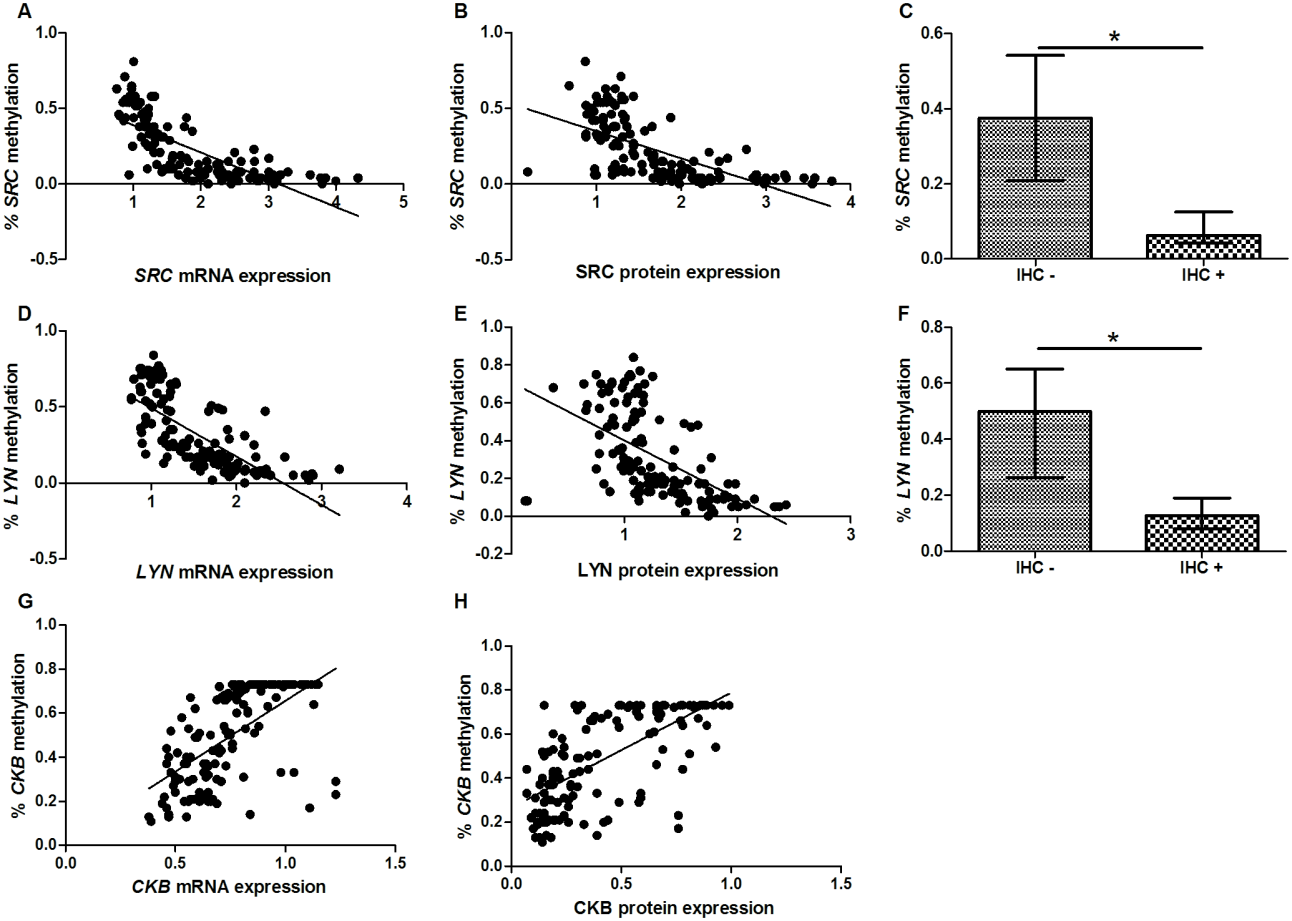
Kinases expression regulation by DNA methylation. A) *SRC* mRNA expression; B) SRC protein expression; C) SRC immunoreactivity; D) *LYN* mRNA expression; E) LYN protein expression; F) LYN protein immunoreactivity; G) *CKB* mRNA expression; H) CKB protein expression. Protein and mRNA expression were determined by Western-blot and RT-qPCR analysis, respectively. In these analyses, the expression in gastric tumors was normalized by matched non-neoplastic gastric tissue. *Significant difference between groups by χ^2^ (for analysis involving IHC data) or Mann-Whitney tests (p < 0.05). IHC–: cases without protein immunoreactivity; IHC+: cases presenting protein immunoreactivity.

We observed that both the mRNA and protein expression of SRC (p < 0.001, ρ = -0.834; p < 0.001, ρ = -0.718; respectively; Fig 6A and 6B) and LYN (p < 0.001, ρ = -0.792; p < 0.001, ρ = -0.654; respectively; Fig 6D and 6E) was inversely correlated to the percentage of promoter methylation. Moreover, tumors with SRC [0.062 (0.08) *versus* 0.375 (0.33); p < 0.001; Mann-Whitney test; Fig 6C] and LYN [0.127 (0.11) *versus* 0.500 (0.39); p < 0.001; Fig 6F] immunoreactivity presented lower percentage of methylation than tumor lacking this protein immunoreactivity.

Concerning CKB regulation, a direct correlation was observed between the percentage of methylation and the CKB mRNA and protein expression (p < 0.001, ρ = 0.684; p < 0.001, ρ = 0.686; respectively; Fig 6G and 6H). Interestingly, increased CKB protein and mRNA expression was observed in tumors with a hypomethylated *CKB* promoter compared with tumors with a partially methylated promoter (p = 0.015, p = 0.008, respectively; Mann-Whitney test followed by Bonferroni corrections; S1 Fig). However, tumors with a hypermethylated *CKB* promoter also presented increased protein and mRNA expression compared with tumors with a partially methylated promoter (p < 0.001, for both comparisons).

### Methylation of kinase promoters and clinicopathological variables

In non-neoplastic gastric mucosa, the percentage of *SRC* (p = 0.010, ρ = -0.218) and *LYN* (p = 0.003, ρ = -0.248) methylation was (weakly) inversely correlated with the age of patients at surgery, though no other association was observed between the percentage of methylation and gender, *H*. *pylori* and EBV infection in the non-neoplastic samples (p > 0.05; Mann-Whitney test).

Table 4 shows the associations between the percentage of methylation in GC samples and clinicopathological characteristics. In neoplastic samples, the percentage of *SRC* (p = 0.002, ρ = -0.267), *LYN* (p = 0.014, ρ = -0.208) and *CKB* (p = 0.024, ρ = -0.192) methylation was (weakly) inversely correlated with the age of patients at surgery. Moreover, the percentage of *SRC* (p = 0.002), *LYN* (p = 0.015) and *CKB* (p = 0.024) methylation was lower in late-onset than in early-onset GC samples. Additionally, we observed that SRC methylation was lower in non-cardia GC in relation to cardia GC (p = 0.028).

**Table 4:** Clinicopathological variables and kinases methylation in gastric cancer.

Variable	N	% *SRC* methylation		% *LYN* methylation		% *CKB* methylation	
		Median (IQR)	p-value	Median (IQR)	p-value	Median (IQR)	p-value
**Gender**							
Female	50	0.13 (0.31)	0.523	0.23 (0.45)	0.424	0.51 (0.42)	0.804
Male	88	0.13 (0.36)		0.24 (0.39)		0.51 (0.42)	
**Onset**							
< 45 years	35	0.31 (0.44)	0.002*	0.28 (0.48)	0.015*	0.63 (0.30)	0.024*
≥ 45 years	103	0.10 (0.31)		0.19 (0.39)		0.50 (0.41)	
**Tumor location**							
Cardia	52	0.11 (0.30)	0.028*	0.19 (0.38)	0.184	0.49 (0.44)	0.076
Non-cardia	82	0.26 (0.41)		0.32 (0.43)		0.53 (0.40)	
**Histological type**							
Diffuse	64	0.19 (0.35)	0.182	0.26 (0.38)	0.579	0.49 (0.46)	0.309
Intestinal	74	0.10 (0.33)		0.21 (0.41)		0.52 (0.39)	
**Stage**							
Early	12	0.41 (0.44)	0.016*	0.52 (0.49)	0.004*	0.73 (0.24)	0.006*
Advanced	126	0.12 (0.33)		0.21 (0.38)		0.50 (0.42)	
**Tumor invasion**							
T1/T2	43	0.31 (0.42)	<0.001*	0.55 (0.46)	<0.001*	0.73 (0.29)	<0.001*
T3/T4	95	0.10 (0.27)		0.17 (0.34)		0.43 (0.42)	
**Lymph node metastasis**							
Absent	16	0.51 (0.16)	<0.001*	0.69 (0.12)	<0.001*	0.73 (0.07)	<0.001*
Present	122	0.10 (0.27)		0.19 (0.32)		0.45 (0.41)	
**Distant metastasis**							
Absent	70	0.37 (0.30)	<0.001*	0.52 (0.32)	<0.001*	0.72 (0.08)	<0.001*
Present	68	0.06 (0.04)		0.12 (0.10)		0.33 (0.22)	
***H*. *pylori***							
Negative	14	0.12 (0.49)	0.520	0.29 (0.46)	0.519	0.56 (0.31)	0.310
Positive	124	0.13 (0.31)		0.24 (0.38)		0.50 (0.43)	
**CagA**							
Negative	49	0.10 (0.32)	0.659	0.25 (0.43)	0.774	0.50 (0.40)	0.588
Positive	89	0.15 (0.35)		0.24 (0.38)		0.52 (0.43)	
**EBV**							
Negative	117	0.15 (0.31)	0.605	0.25 (0.39)	0.144	0.52 (0.42)	0.402
Positive	21	0.12 (0.41)		0.13 (0.35)		0.40 (0.44)	

*Significantly difference between groups by Mann-Whitney test (p < 0.05)

N: number of samples; IQR: interquartile range; EBV: *Epstein-Barr virus*

Reduced percentage of *SRC*, *LYN* and *CKB* methylation were associated with advanced stage, deeper tumor invasion, and the presence of lymph node and distant metastases (p < 0.05, for all comparisons; Table 4). The comparison of *SRC* and *LYN* methylation pattern by MSP and clinicopathological characteristics presented similar results (S2 Table). However, partial methylation of *CKB* by MSP was also more frequently found than hypermethylation in T3/T4 tumors (p = 0.008; S2 Table). *CKB* partial methylation was also more frequent than hypermethylation (p < 0.001) and hypomethylation (p = 0.009; S2 Table) in tumors from individuals with distant metastasis in relation to tumors from individuals without distant metastasis.

A gradual decrease in the percentage of *SRC* and *LYN* methylation was observed corresponding to the tumor stage (p < 0.008, for most of the comparisons; Mann-Whitney test followed by Bonferroni correction; Fig 3C and 3F). With regard to *CKB* methylation, 12 of 14 (85.7%) of the samples of GC in the stage I presented 73.3% of methylation. The percentage of *CKB* methylation was higher in stage I than in stages II, III and IV (p < 0.008, for most of these comparisons; Fig 3I). Conversely, the percentage of *CKB* methylation was significantly reduced in the stage IV in relation to the other stages (p < 0.008, for these comparisons; Fig 3I).

### Kinases and MYC relationships

We examined MYC immunoreactivity, mRNA expression and methylation status data for a set of 49 of the studied pairs of neoplastic and non-neoplastic samples. [18]

MYC immunoreactivity was detected in 38 (77.6%) tumors. The immunoreactivity of MYC was associated with the immunoreactivity of SRC (p < 0.001, by χ^2^ test) and LYN (p < 0.004, by χ^2^ test), with 2 (4.1%) GC samples presenting only kinase immunoreactivity and 9 (18.4%) GC samples without MYC or kinase immunoreactivity.

**Fig 7:**
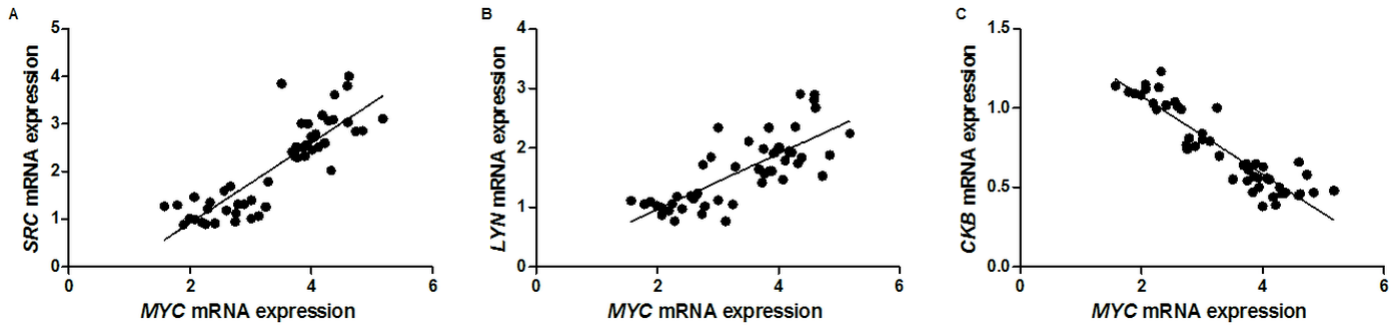
Correlation between *MYC* and kinases mRNA expression. A) *MYC* and *SRC*; B) *MYC* and *LYN*; C) *MYC* and *CKB*.

The mRNA level of *MYC* was increased at least 1.5-fold in all GC samples in relation to matched non-neoplastic gastric samples. In addition, a direct correlation was observed between the mRNA expression of *MYC* and *SRC* (p < 0.001, ρ = 0.856; Fig 7A) and *MYC* and *LYN* (p < 0.001, ρ = 0.763; Fig 7B). In contrast, an inverse correlation was observed between *MYC* and *CKB* mRNA expression (p < 0.001, ρ = -0.890; Fig 7C).

In non-neoplastic samples, a direct correlation was observed between the *MYC* and *SRC* methylation patterns (p < 0.001, ρ = 0.486) and between *MYC* and *LYN* methylation patterns (p < 0.001, ρ = 0.647) by MSP. In addition, an inverse correlation was detected between *MYC* and *CKB* (p< 0.001, ρ = -0.320). However, no correlation was observed between *MYC* and the three studied kinases in the GC samples: *MYC* and *SRC* (p = 0.626, ρ = -0.071); *MYC* and *LYN* (p = 0.724, ρ = 0.052); *MYC* and *CKB* (p = 0.820, ρ = -0.039).

## Discussion

Kinases are the most intensively studied category of protein drug targets in current pharmacological research, as evidenced by the vast number of kinase-targeting agents enrolled in active clinical trials [4]. In the present study, we evaluated the role of CKB and of two members of the SRC family of kinases, SRC and LYN. We observed that the SRC and LYN kinases were upregulated in approximately 50% of GC samples. Aberrant SRC activity has already been observed in several human cancers, including GC [7–9]. In addition, LYN overexpression has been reported in several cancers, such as chronic myelogenous leukemia [26], colorectal cancer [27], breast cancer [28], prostate cancer [29], oral cancer [30], renal cancer [31] and Ewing’s sarcoma [32]; nonetheless, no previous study has evaluated the role of LYN in gastric carcinogenesis. Our results suggest that SRC and LYN may be targets of anticancer therapies in GC patients presenting elevated expression of these kinases.

SRC immunoreactivity or elevated protein and mRNA expression was associated with late onset, an advanced stage, deeper tumor extension and the presence of metastasis. Yang et al. demonstrated that SRC regulates migration and invasion in a GC cell line (BGC-823) following treatment of these cells with the SRC inhibitors PP2 and SU6656, which is in part in agreement with our findings in primary GC samples [56]. Moreover, as for SRC, LYN may also have a role in gastric tumor invasiveness, metastasis, and thus aggressiveness. These associations have been suggested for other cancers. LYN upregulation was associated with colorectal tumor grade, stage, and lymph node and distant metastases [27]. In addition, the inhibition of LYN was able to decrease primary tumor growth, reduce metastases in an in vivo model of Ewing’s sarcoma, and decrease the invasive capacity of Ewing’s sarcoma cells in vitro [32].

The mitogenic function of SRC is, at least in part, mediated by the induction of MYC [12, 13]. Here, we report a direct correlation between SRC and LYN expression, as well as between the expression of these kinases and that of MYC. As for SRC and LYN, MYC immunoreactivity or elevated mRNA expression was previously associated with late onset, advanced stage, deeper tumor extension and the presence of metastasis [18]. We also previously described MYC deregulation in preneoplastic gastric lesions [15, 19, 57]. Therefore, our results suggest that the observed associations among SRC, LYN and MYC might be necessary for gastric carcinogenesis progression.

In our study, CKB downregulation was observed in GC samples. *CKB* downregulation was previously detected in a transcriptomic study [43], however, further validation by other methodologies was lacking. This enzyme is overexpressed in a wide variety of cancers [37, 58, 59], with the exception of colon cancer [60, 61]. Li et al. showed that CKB knockdown inhibits ovarian cancer progression by decreasing glycolysis [60]. Our previous proteomic study suggested the presence of a Warburg effect in GC [40], and we also reported the upregulation of the *MYC* oncogene [14–18], which appears to contribute to this effect [39]. Therefore, the downregulation of CKB and its strong inverse correlation with MYC expression is not in agreement with the role of CKB in the regulation of glycolysis in gastric carcinogenesis [40].

Mooney et al. suggested that ATP storage is not the most important function of CKB in colon cancer, in which the expression of CKB protein and mRNA are downregulated [61]. These authors showed that the overexpression of CKB-C283S, a dominant-negative construct with effects similar to CKB downregulation, appears to promote the epithelial-to-mesenchymal transition in colon cancer [61]. In addition, the authors showed that although CKB expression may be advantageous to the formation of a solid tumor, it appears to be a hindrance to the metastatic potential of colon cancer cells. Similar to the findings of Mooney et al. [61], our results also showed that reduced CKB in GC may have a role in tissue invasion and metastasis. Moreover, Mooney et al. also showed that colon cancer cells overexpressing CKB-C283S and cultured in medium without glucose presented higher expression of MYC than cells with a wild-type CKB construct [61]. In the present study, we detected a strong inverse correlation between CKB and MYC. Thus, an inverse correlation between CKB and MYC might also be necessary for GC progression.

Interestingly, increased SRC and LYN expression and reduced CKB expression were also associated with late-onset GC. Clinicopathological differences between early-onset and late-onset GC have been described [62–64], but little is known about the genetic and epigenetic changes associated with the age of onset of GC [65]. Buffart et al. previously demonstrated that young and old patients belong to groups with different genomic profiles [66]. The deregulation of the three studied kinases highlights the heterogeneity of GC.

DNA methylation of CpG islands plays a crucial role in the regulation of gene expression. Our group previously reported alterations in the DNA methylation pattern of several oncogenes and tumor suppressor genes in GC samples of individuals from Northern Brazil [18, 67–73]. According to CpG Island Searcher, the *SRC* and *LYN* genes contain a CpG island of more than 1 kb (http://cpgislands.usc.edu/; version: 10/29/04). The first CpG island in the *SRC* gene is between intron 1 and intron 2, and the *LYN* gene has a CpG island in its promoter, exon 1 and part of intron 1. Among the three studied kinases, *CKB* has the largest CpG island, almost 3 kb, located between its promoter and intron 3. To the best of our knowledge, no previous study has evaluated the methylation patterns of these kinases in gastric tissue samples.

In the present study, we observed that the *SRC*, *LYN* and *CKB* methylation patterns were altered in GC. Reduced *SRC* and *LYN* methylation was detected in GC samples. The reduced *SRC* and *LYN* methylation was associated with increased levels of mRNA and protein expression, suggesting that DNA methylation is involved in regulating the expression of these kinases. Moreover, patients with tumors presenting loss of *SRC* and *LYN* methylation also exhibited altered methylation for these genes in non-neoplastic gastric mucosa, albeit at a lower level. Although further investigation of premalignant GC is necessary, this finding suggests that individuals with the loss of *SRC* and *LYN* methylation in the gastric mucosa may have a higher risk for GC.

The identification of specific DNA methylation patterns may help in the classification of GC and could be associated with specific clinical outcomes. Here, we report that reduced *SRC* and *LYN* methylation was associated with advanced stage GC, deeper tumor invasion and with lymph node or distant metastasis. These findings support the hypothesis that DNA methylation is involved in *SRC* and *LYN* regulation because we also observed that the elevated expression of theses kinases may have a role in GC invasiveness and metastasis. DNA is a stable molecule, and the detection of DNA methylation, especially by the MSP assay (a qualitative method), may be readily used as an approach for GC prognosis in the clinical routine. Therefore, analysis of the *SRC* and *LYN* methylation may help in determining GC prognosis.

Reduced *SRC* and *LYN* methylation and their increased expression were associated with late-onset GC. The incidence of GC increases with age, highlighting the association between age-related methylation and GC development [71, 74].

In contrast, the percentage of *CKB* methylation was higher in GC samples than in non-neoplastic gastric samples. However, increased percentage of CpG methylated sites in *CKB* promoter were correlated with increased mRNA and protein expression. On the other hand, tumors with partial methylation of *CKB* presented reduced protein and mRNA expression compared to tumors with hypermethylated and hypomethylated *CKB*. Ishikawa et al. [44] previously evaluated *CKB* methylation patterns in seven GC cell lines and other solid tumor cell lines and observed that *CKB* promoter methylation was not associated with mRNA expression. We hypothesized that DNA methylation of other CpGs, as well as other genetic and epigenetic mechanisms, may also have a role in *CKB* gene expression. Moreover, post-transcriptional mechanisms may be involved in CKB regulation in gastric carcinogenesis because we observed that the frequency of GC samples presenting reduced CKB protein expression was higher than those presenting reduced mRNA expression.

Although reduced CKB expression was associated with a poor prognosis and late-onset GC, reduced *CKB* methylation was associated with advanced stage, deeper tumor invasion, lymph node or distant metastasis and with late-onset GC. This finding is in agreement with the observation of a direct correlation between CKB expression and methylation and reinforces that further investigation are still necessary to understand the role of *CKB* methylation in gastric carcinogenesis. However, *CKB* methylation might contribute to GC cell migration and invasion.

In conclusion, our study provides a basis for the development of a biomarker for the prognosis of GC. Expression of SRC, LYN and CKB in gastric cancer is significantly associated with tumor invasion and lymph node and distant metastases, as well as with MYC expression, which is also a possible biomarker for GC. In addition, these three kinases appear to be regulated, at least in part, by DNA methylation in GC. SRC, LYN and CKB proteins or DNA methylation could serve as markers for predicting tumor progression and target in anti-cancer strategies.

## Supporting Information

S1 Fig Schematic flowchart of the study design. GC: gastric cancer samples; FFPE: formalin-fixed and paraffin-embedded; EBV: Epstein-Barr virus; IHC: immunohistochemistry; HP: *Helicobacter pylori*; WB: Western blot; MSP: methylation-specific PCR; BSP: bisulfite sequencing PCR; RT-qPCR: reverse transcription quantitative PCR.
(TIF)

S2 Fig Association between DNA methylation pattern by methylation-specific PCR and kinases expression. A) SRC immunoreactivity; B) SRC protein expression; C) SRC mRNA expression; D) LYN protein immunoreactivity; E) LYN protein expression; F) LYN mRNA expression; G) CKB immunoreactivity; H) CKB protein expression; I) CKB mRNA expression. Protein and mRNA expression were determined by Western-blot and RT-qPCR analysis, respectively. In these analyses, the expression in gastric tumors was normalized by matched non-neoplastic gastric tissue. *Significant difference between groups by χ2 (for analysis involving IHC data) or Mann-Whitney tests followed by Bonferroni corrections for multiple comparison analysis (p < 0.0167); **Significant difference between groups by χ2 (for analysis involving IHC data) or Mann-Whitney tests followed by Bonferroni corrections for multiple comparison analysis (p < 0.001). IHC+: cases presenting protein immunoreactivity; IHC–: cases without protein immunoreactivity.
(TIF)

S1 File Screening of kinases using capture compound mass spectrometry. (DOCX)

S1 Table Kinases in gastric cancer by capture compound methodology. (DOCX)

S2 Table Clinicopathological variables and kinases methylation pattern by methylation-specific PCR in gastric cancer. (DOCX)
