# Supplementary figures and images for: Deregulated Expression of SRC, LYN and CKB Kinases by DNA Methylation and Its Potential Role in Gastric Cancer Invasiveness and Metastasis
Source: PLoS One. 2015 Oct 13;10(10):e0140492. doi: 10.1371/journal.pone.0140492 (PMC4604160; doi:10.1371/journal.pone.0140492)

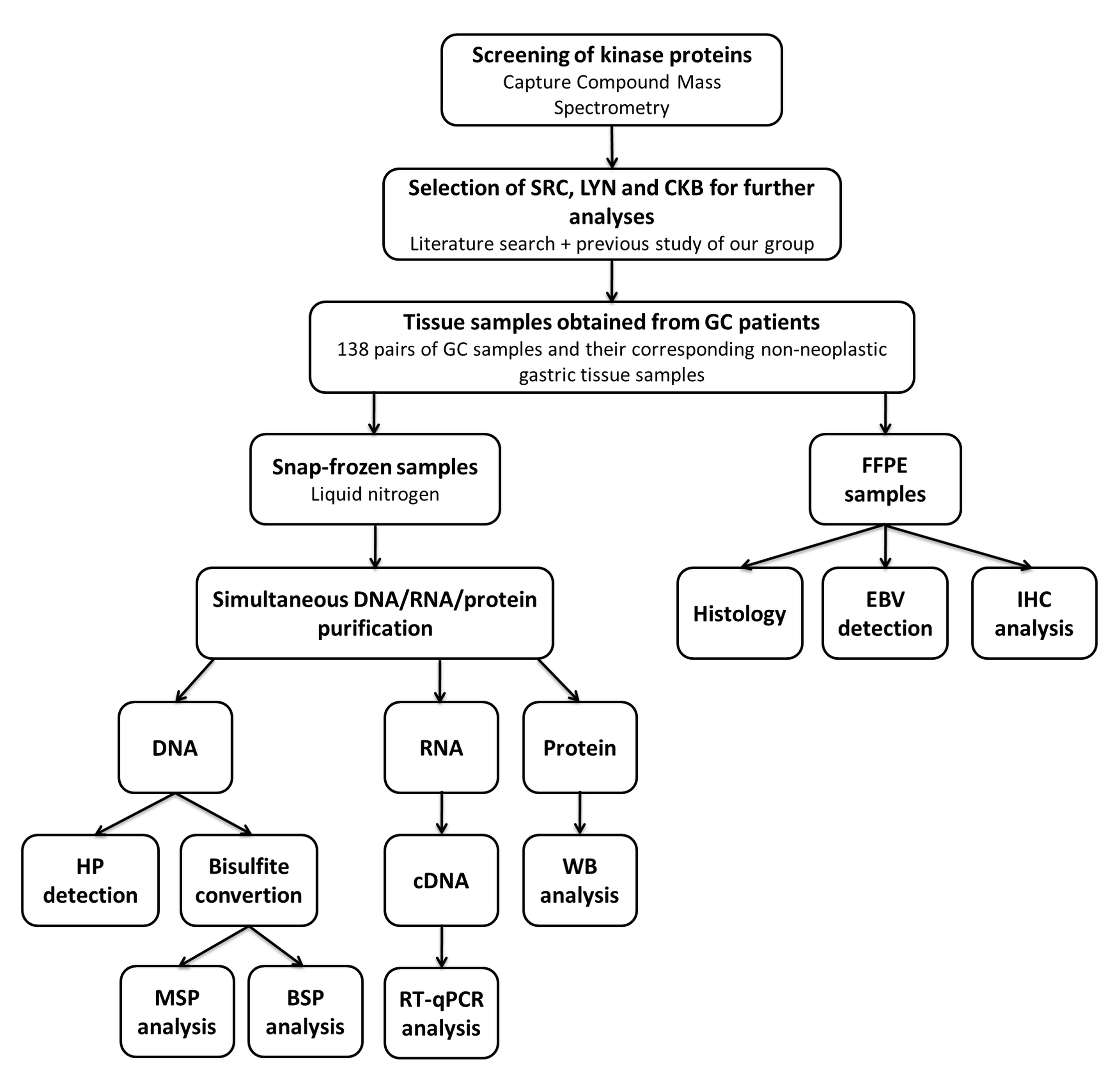

Supplement: S1 Fig — GC: gastric cancer samples; FFPE: formalin-fixed and paraffin-embedded; EBV: Epstein-Barr virus; IHC: immunohistochemistry; HP: Helicobacter pylori; WB: Western blot; MSP: methylation-specific PCR; BSP: bisulfite sequencing PCR; RT-qPCR: reverse transcription quantitative PCR. (TIF) [file pone.0140492.s001.tif]

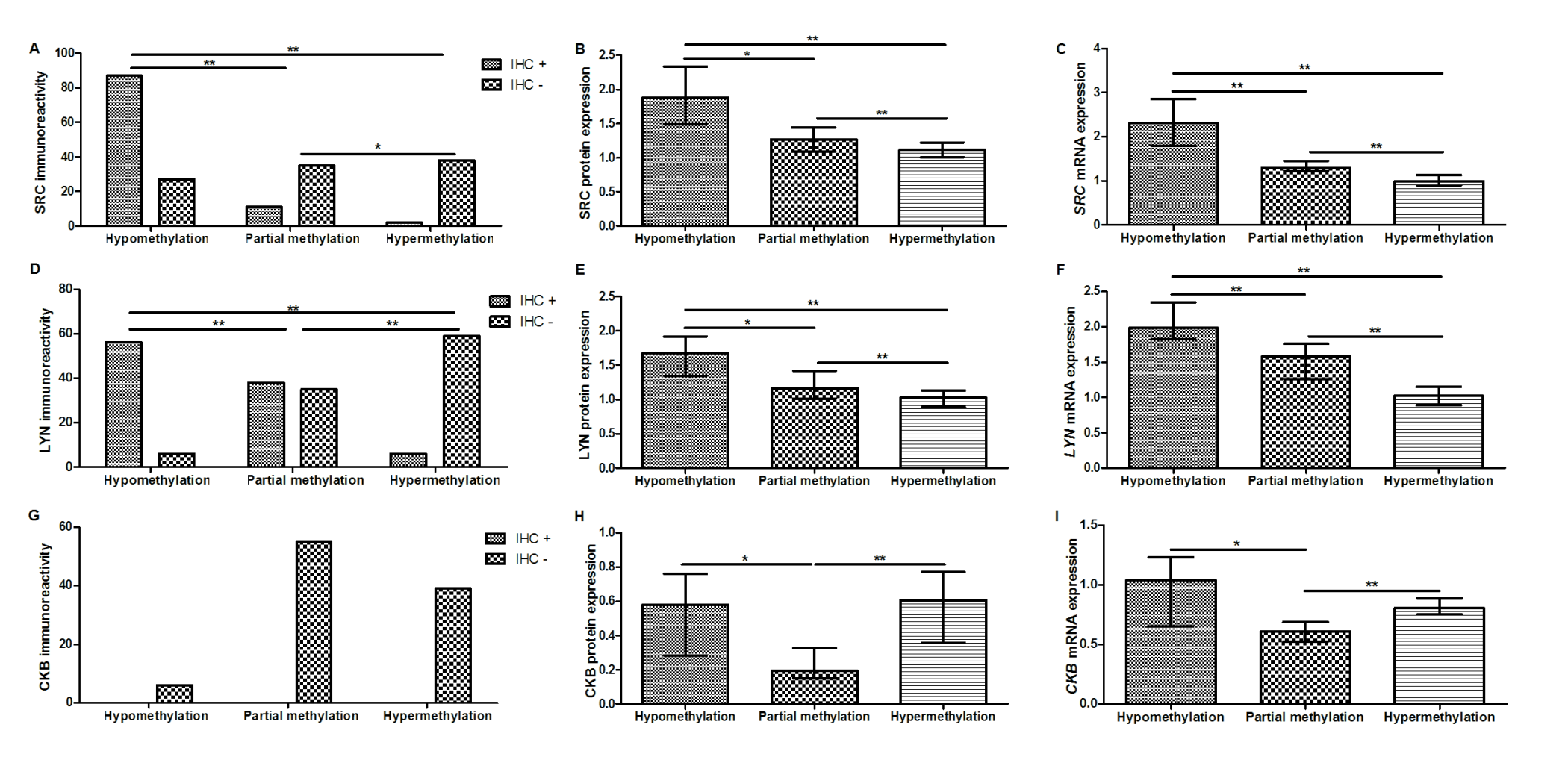

Supplement: S2 Fig — A) SRC immunoreactivity; B) SRC protein expression; C) SRC mRNA expression; D) LYN protein immunoreactivity; E) LYN protein expression; F) LYN mRNA expression; G) CKB immunoreactivity; H) CKB protein expression; I) CKB mRNA expression. Protein and mRNA expression were determined by Western-blot and RT-qPCR analysis, respectively. In these analyses, the expression in gastric tumors was normalized by matched non-neoplastic gastric tissue. *Significant difference between groups by χ2 (for analysis involving IHC data) or Mann-Whitney tests followed by Bonferroni corrections for multiple comparison analysis (p < 0.0167); **Significant difference between groups by χ2 (for analysis involving IHC data) or Mann-Whitney tests followed by Bonferroni corrections for multiple comparison analysis (p < 0.001). IHC+: cases presenting protein immunoreactivity; IHC–: cases without protein immunoreactivity. (TIF) [file pone.0140492.s002.tif]
