# Supplementary material for: Deregulated Expression of SRC, LYN and CKB Kinases by DNA Methylation and Its Potential Role in Gastric Cancer Invasiveness and Metastasis
Source: PLoS One. 2015 Oct 13;10(10):e0140492. doi: 10.1371/journal.pone.0140492 (PMC4604160; doi:10.1371/journal.pone.0140492)
Supplement: S1 File — (DOCX) [file pone.0140492.s003.docx]

**S1 File. Screening of kinases using capture compound mass spectrometry**

A 100-mg sample of three gastric cancer tissue specimens from the same population described in the methodological section were lysed in 1 ml of buffer composed of 20 mM HEPES/NaOH pH 7.4, 250 mM sucrose, 5 mM MgCl_2_, 2 mM ß-mercaptoethanol and Protease Inhibitor cocktail (#P8340, Roche, USA) using Tissue Ruptor (Qiagen, USA). Then, 0.5% of CHAPS was added, and the lysates were homogenized for 1 hour at room temperature. The debris was removed by centrifugation, and the protein concentration was determined by the method of Bradford (Sigma-Aldrich, USA). The samples were concentrated using Millipore Microcon Centrifugal Filter Devices YM-10 (10 kDa cut-off; Millipore, USA) to a concentration of at least 3.2 µg/µl. A total of 140 µg of protein was used per experiment.

The experiments were carried out using proprietary technology from Caprotec for capture compound mass spectrometry. [^1^](#_ENREF_1)^,^ [^2^](#_ENREF_2) A staurosporine-based stauro-capture compound was used for the enrichment of kinases and other ATP-binding proteins from the proteins purified from the three tumor samples. After enzymatic digestion, the captured proteins were analyzed by liquid chromatography tandem mass spectrometry (LC-MS/MS) according to Luo et al. [^3^](#_ENREF_3) Peptides were injected into an Ultimate 3000 liquid chromatography system (Dionex, Germany) equipped with a nanoflow Biosphere C_18_ trap-column (5 µm, 120 Å, 20 x 0.1 mm, nanoseparations, The Netherlands) and a nanoflow Biosphere C_18_ analytical column (5 µm, 120 Å, 100 x 0.075 mm, nanoseparations, The Netherlands). The mass analysis was performed using an LTQ Orbitrap XL mass spectrometer (Thermo Fisher Scientific, Germany) and a nanoelectrospray ion source (Proxeon Biosystems A/S, Denmark). The MS analysis was performed in the data-dependent mode to automatically switch between full-scan MS from m/z 300-2000 in the Orbitrap analyzer and MS/MS acquisition in the linear ion trap. The 5 most intense peptide ions were sequentially isolated to a target value of 10.000 and fragmented in the linear ion trap using collision-induced dissociation. All MS/MS samples were analyzed using SEQUEST implemented in BioworksBrowser 3.3.1 SP1 (Thermo Fisher Scientific, Germany) and X!Tandem. [^4^](#_ENREF_4) A label-free quantification was performed using Andromeda implemented in MaxQuant (www.maxquant.org; release 1.2.2.4). For the analysis of all MS/MS samples, an automated database search against the human UniProtKB/Swiss-Prot database (release 2011_01) was performed with 6 ppm precursor tolerance, 0.5 Da fragment ion tolerance, full trypsin specificity allowing for up to 2 missed cleavages and methionine oxidation as a variable modification. The maximum false discovery rates were set to 0.01 at both the protein and peptide levels, the maximum posterior error probability (PEP) was set to 1, and 6 amino acids were required as the minimum peptide length. The label-free quantification option was selected with a maximal retention time window of 2 min for alignment between LC-MS/MS runs.

**References**

1. Koster H, Little DP, Luan P, Muller R, Siddiqi SM, Marappan S, Yip P. Capture compound mass spectrometry: a technology for the investigation of small molecule protein interactions. Assay Drug Dev Technol 2007; 5:381-90.

2. Dalhoff C, Huben M, Lenz T, Poot P, Nordhoff E, Koster H, Weinhold E. Synthesis of S-adenosyl-L-homocysteine capture compounds for selective photoinduced isolation of methyltransferases. ChemBioChem 2010; 11:256-65.

3. Luo Y, Blex C, Baessler O, Glinski M, Dreger M, Sefkow M, Koster H. From PKA to HCN: The cAMP-capture compound mass spectrometry as a novel tool for targeting cAMP binding proteins. Mol Cell Proteomics 2009.

4. Craig R, Beavis RC. TANDEM: matching proteins with tandem mass spectra. Bioinformatics 2004; 20:1466-7.
