# Supplementary material for: Deregulated Expression of SRC, LYN and CKB Kinases by DNA Methylation and Its Potential Role in Gastric Cancer Invasiveness and Metastasis
Source: PLoS One. 2015 Oct 13;10(10):e0140492. doi: 10.1371/journal.pone.0140492 (PMC4604160; doi:10.1371/journal.pone.0140492)
Supplement: S1 Table — (DOCX) [file pone.0140492.s004.docx]

**S1 Table. Kinases in gastric cancer by capture compound methodology**

| Gene Names | Molecular Weight [kDa] | PEP | Unique Peptides | Intensity | | |
| --- | --- | --- | --- | --- | --- | --- |
|  |  |  |  | Tumor #1 | Tumor #2 | Tumor #3 |
| LYN;HCK | 58,573 | 1,78E-07 | 1 | 1363900 | 533120 | 154380 |
| PFKM;PFKX | 85,182 | 1,16E-08 | 2 | 0 | 238090 | 258940 |
| CKB;CKBB | 42,644 | 6,76E-104 | 8 | 0 | 6539400 | 14980000 |
| SRC;SRC1 | 60,588 | 1,24E-34 | 6 | 1343100 | 3278500 | 362640 |
| OIP3;PK2;PK3;PKM;PKM2 | 57,936 | 1,03E-259 | 17 | 4591300 | 3302800 | 4723400 |
| PFKL | 85,018 | 3,33E-87 | 1 | 289740 | 381410 | 370350 |
| KIAA1477;MARK;MARK1;EMK1;MARK2;CTAK1;EMK2;MARK3;KIAA1860;MARK4;MARKL1 | 89,002 | 1,01E-03 | 1 | 526610 | 231810 | 143900 |
| CSK | 50,704 | 8,17E-07 | 2 | 1228600 | 84371 | 0 |
| ISPK1;MAPKAPK1B;RPS6KA3;RSK2 | 83,735 | 1,36E-22 | 4 | 1545800 | 172390 | 141510 |
| PFKF;PFKP | 85,595 | 1,16E-144 | 8 | 132870 | 575360 | 380550 |
| FAK;FAK1;PTK2;FAK2;PTK2B;PYK2;RAFTK | 119,23 | 4,14E-02 | 1 | 401640 | 0 | 0 |
| PRKCD | 77,504 | 3,03E-22 | 2 | 816060 | 649990 | 210460 |
| MST1;STK4;MST2;STK3 | 55,63 | 6,15E-06 | 2 | 124020 | 392440 | 0 |
| AMPK1;PRKAA1;AMPK;AMPK2;PRKAA2 | 65,522 | 1,47E-14 | 2 | 126110 | 558040 | 165440 |
| ILK;ILK1;ILK2 | 51,419 | 1,77E-19 | 7 | 80359 | 4374100 | 2538100 |
| CAMK2D;CAMKD | 59,151 | 9,24E-63 | 5 | 3568200 | 3850600 | 1757900 |
| PRKG1;PRKG1B;PRKGR1A;PRKGR1B | 76,364 | 8,33E-66 | 1 | 1254800 | 1115500 | 0 |
| CAMK1 | 41,337 | 6,56E-03 | 1 | 0 | 412360 | 97294 |
| MLCK;MYLK | 210,71 | 1,71E-121 | 13 | 5972000 | 46032000 | 49012000 |
| PAK1;PKN;PKN1;PRK1;PRKCL1 | 104,67 | 1,05E-07 | 3 | 192230 | 653820 | 110200 |
| AAK1;KIAA1048 | 103,88 | 6,67E-15 | 1 | 551200 | 649790 | 0 |
| NAGK | 37,375 | 8,95E-02 | 1 | 542030 | 0 | 0 |

PEP: posterior error probality, which is a quality score of the protein identification (similar to a p‐value).
