# Supplementary material for: Deregulated Expression of SRC, LYN and CKB Kinases by DNA Methylation and Its Potential Role in Gastric Cancer Invasiveness and Metastasis
Source: PLoS One. 2015 Oct 13;10(10):e0140492. doi: 10.1371/journal.pone.0140492 (PMC4604160; doi:10.1371/journal.pone.0140492)
Supplement: S2 Table — (DOCX) [file pone.0140492.s005.docx]

**S2 Table. Clinicopathological variables and kinases methylation pattern by methylation-specific PCR in gastric cancer**

| Variable | N | *SRC* | | | | *LYN* | | | | *CKB* | | | |
| --- | --- | --- | --- | --- | --- | --- | --- | --- | --- | --- | --- | --- | --- |
|  |  | Hypermethylated [N(%)] | Partial-methylated [N(%)} | Hypomethylated [N(%)] | p-value | Hypermethylated [N(%)] | Partial-methylated [N(%)} | Hypomethylated [N(%)] | p-value | Hypermethylated [N(%)] | Partial-methylated [N(%)} | Hypomethylated [N(%)] | p-value |
| **Gender** |  |  |  |  |  |  |  |  |  |  |  |  |  |
| Female | 50 | 10 (20) | 10 (20) | 30 (60) | 0.865 | 18 (36) | 16 (32) | 16 (32) | 0.709 | 16 (36.4) | 26 (59.1) | 2 (4.5) | 0.794 |
| Male | 88 | 16 (18.2) | 21 (23.9) | 51 (58) |  | 28 (32.2) | 34 (39.1) | 25 (28.7) |  | 32 (40.5) | 42 (53.2) | 5 (6.3) |  |
| **Onset** |  |  |  |  |  |  |  |  |  |  |  |  |  |
| < 45 years | 35 | 10 (28.6) | 6 (17.1) | 19 (54.3) | 0.212 | 19 (54.3) | 8 (22.9) | 8 (22.9) | 0.010* | 16 (53.3) | 14 (46.7) | 0 (0) | 0.087 |
| ≥ 45 years | 103 | 15 (15.5) | 25 (24.3) | 62 (60.2) |  | 27 (26.5) | 33 (32.4) | 42 (41.2) |  | 32 (34.4) | 54 (58.1) | 7 (7.5) |  |
| **Tumor location** |  |  |  |  |  |  |  |  |  |  |  |  |  |
| Cardia | 52 | 10 (19.2) | 16 (30.8) | 26 (50) | 0.162 | 21 (41.2) | 17 (33.3) | 13 (25.5) | 0.341 | 19 (41.3) | 25 (54.3) | 2 (4.3) | 0.843 |
| Non-cardia | 82 | 16 (18.6) | 15 (17.4) | 55 (64) |  | 25 (29.1) | 33 (38.4) | 28 (32.6) |  | 29 (37.7) | 43 (55.8) | 5 (6.5) |  |
| **Histological type** |  |  |  |  |  |  |  |  |  |  |  |  |  |
| Diffuse | 64 | 13 (20.3) | 8 (12.5) | 43 (67.2) | 0.032* | 19 (29.7) | 22 (34.4) | 23 (35.9) | 0.343 | 22 (39.3) | 33 (58.9) | 1 (1.8) | 0.223 |
| Intestinal | 74 | 13 (17.6) | 23 (31.1) | 38 (51.4) |  | 27 (37) | 28 (38.4) | 18 (24.7) |  | 26 (38.8) | 35 (52.2) | 6 (9) |  |
| **Stage** |  |  |  |  |  |  |  |  |  |  |  |  |  |
| Early | 12 | 7 (58.3) | 1 (8.3) | 4 (33.3) | 0.001* | 10 (83.3) | 1 (8.3) | 1 (8.3) | 0.001* | 5 (50) | 5 (50) | 0 (0) | 0.603 |
| Advanced | 126 | 19 (15.1) | 30 (23.8) | 77 (61.1) |  | 36 (28.8) | 49 (39.2) | 40 (32) |  | 43 (38.1) | 63 (55.8) | 7 (6.2) |  |
| **Tumor invasion** |  |  |  |  |  |  |  |  |  |  |  |  |  |
| T1/T2 | 43 | 17 (39.5) | 8 (18.6) | 18 (41.9) | <0.001* | 26 (60.5) | 14 (32.6) | 3 (7) | <0.001* | 20 (57.1) | 12 (34.3) | 3 (8.6) | 0.013* |
| T3/T4 | 95 | 9 (9.5) | 23 (24.2) | 63 (66.3) |  | 20 (21.3) | 36 (38.3) | 38 (40.4) |  | 28 (31.8) | 56 (63.6) | 4 (4.5) |  |
| **Lymph node metastasis** |  |  |  |  |  |  |  |  |  |  |  |  |  |
| Absent | 16 | 12 (75) | 3 (18.8) | 1 (6.3) | <0.001* | 16 (100) | 0 (0) | 0 (0) | <0.001* | 7 (63.6) | 3 (27.3) | 1 (9.1) | 0.147 |
| Present | 122 | 14 (11.5) | 28 (23) | 80 (65.6) |  | 30 (24.8) | 50 (41.3) | 41 (33.9) |  | 41 (36.6) | 65 (58) | 6 (5.4) |  |
| **Distant metastasis** |  |  |  |  |  |  |  |  |  |  |  |  |  |
| Absent | 70 | 25 (35.7) | 27 (38.6) | 18 (25.7) | <0.001* | 39 (56.5) | 23 (33.3) | 7 (10.1) | <0.001* | 37 (67.3) | 13 (23.6) | 5 (9.1) | <0.001* |
| Present | 68 | 1 (1.5) | 4 (5.9) | 63 (92.6) |  | 7 (10.3) | 27 (39.7) | 34 (50) |  | 11 (16.2) | 55 (80.9) | 2 (2.9) |  |
| ***H. pylori*** |  |  |  |  |  |  |  |  |  |  |  |  |  |
| Negative | 14 | 3 (21.4) | 1 (7.1) | 10 (71.4) | 0.348 | 7 (50) | 3 (21.4) | 4 (28.6) | 0.328 | 5 (45.5) | 6 (54.5) | 0 (0) | 0.665 |
| Positive | 124 | 23 (18.5) | 30 (24.2) | 71 (57.3) |  | 39 (31.7) | 47 (38.2) | 37 (30.1) |  | 43 (38.4) | 62 (55.4) | 7 (6.3) |  |
| **CagA** |  |  |  |  |  |  |  |  |  |  |  |  |  |
| Negative | 49 | 10 (20.4) | 7 (14.3) | 32 (65.3) | 0.231 | 17 (35.4) | 17 (35.4) | 14 (29.2) | 0.945 | 18 (43.9) | 20 (48.8) | 3 (7.3) | 0.568 |
| Positive | 89 | 16 (18) | 24 (27) | 49 (55.1) |  | 29 (32.6) | 33 (37.1) | 27 (30.3) |  | 30 (36.6) | 48 (58.5) | 4 (4.9) |  |
| **EBV** |  |  |  |  |  |  |  |  |  |  |  |  |  |
| Negative | 117 | 22 (18.8) | 27 (23.1) | 68 (58.1) | 0.917 | 40 (34.5) | 43 (37.1) | 33 (28.4) | 0.668 | 40 (38.8) | 57 (55.3) | 6 (5.8) | 0.987 |
| Positive | 21 | 4 (19) | 4 (19) | 13 (61.9) |  | 6 (28.6) | 7 (33.3) | 8 (38.1) |  | 8 (40) | 11 (55) | 1 (5) |  |

*Significantly difference between groups by χ^2^ test (p < 0.05). N: number of samples; EBV: *Epstein-Barr virus.*
